# Buffering zone of implantable Collamer lens sizing in V4c

**DOI:** 10.1186/s12886-017-0663-4

**Published:** 2017-12-22

**Authors:** Seung Wan Nam, Dong Hui Lim, Joo Hyun, Eui-Sang Chung, Tae-Young Chung

**Affiliations:** 1 Department of Ophthalmology, Samsung Medical Center, Sungkyunkwan University School of Medicine, #81 Irwon-ro, Gangnam-gu, Seoul, 06351 South Korea; 2 Department of Preventive Medicine, Graduate School, The Catholic University of Korea, Seoul, South Korea; 3 Saevit Eye Hospital, Goyang, South Korea

**Keywords:** Implantable collamer lens, Postoperative vault, Sulcus-to-sulcus diameter

## Abstract

**Background:**

The purpose of this study was to identify factors related to the unexpected vault in V4c implantable collamer lens (ICL; STAAR Surgical) implantation.

**Methods:**

V4c ICLs were implanted in 43 eyes of 43 patients for the correction of myopia. The implanted V4c ICL sizes were determined individually with our previous V4 ICL sizing nomogram based on the sulcus-to-sulcus diameter (STS), and the V4 ICL sizes were then converted to V4c ICL sizes with a size-converting table. We defined the “normal-sizing group” as having a pre-converted ICL size larger than the STS, and the “under-sizing group” as having a pre-converted ICL size smaller than the STS. Refractive outcomes, safety and parameters related to postoperative vault were compared between the two groups.

**Results:**

The value of “actual ICL size – STS” differed significantly between the normal-sizing and under-sizing groups (*p* < 0.001), but postoperative vault did not differ significantly (*p* = 0.442). The demographics, implanted ICL characteristics, effectiveness indexes, safety indexes, and parameters related to postoperative vault did not differ significantly between the two groups (*p* > 0.05). Two patients in the normal-sizing group exhibited over-vaulting; these patients had shallow anterior chambers and were implanted with high-dioptric-power ICLs.

**Conclusions:**

The achievement of acceptable vault in both normal-sizing and under-sizing groups indicates the existence of a buffering zone in V4c ICL sizing. The smaller size of V4c ICLs should be considered in patients susceptible to over-vaulting, such as those with shallow anterior chambers and high-dioptric-power ICLs.

## Background

The advantages of the Visian implantable collamer lens (ICL; STAAR surgical, Monrovia, California, USA), including its favorable outcomes and safety for the correction of myopia, have already been reported elsewhere [1, 2]. However, several complications of ICL implantation have been reported, such as cataracts, secondary glaucoma, and corneal endothelial damage [3, 4]. Postoperative vault, which is the distance between the anterior surface of the crystalline lens and the posterior surface of the ICL, is a determinant of major complications after ICL implantation [5–10]. Many ICL sizing nomograms for achieving acceptable vault have been reported for widely used V4 ICL models [11–14].

The initial V1 ICL model has evolved to the V4c ICL model through the introduction of several design changes to improve visual quality and reduce complications. The differences between V4c and the previous V4 model are that the V4c model has a central hole in the optic and is preserved in balanced salt solution (BSS). These factors may affect the amount of vault [15, 16].

ICL sizing based on sulcus-to-sulcus diameter (STS) method performed by ultrasound biomicroscopy (UBM) is known to be more accurate than the conventional white-to-white corneal diameter (WTW) method because the ICL haptic footplates are located on the ciliary sulcus [17–20]. Therefore, the difference between the ICL size and the STS is the single most important factor in estimation of the ICL vault [12]. In a previous study, we described a V4 ICL sizing nomogram based on the STS that demonstrated good visual and safety outcomes, [12] but we were still achieving unexpectedly high or low vaulting, requiring ICL exchange. Furthermore, according to the manufacturer, the recently developed V4c ICL preserved in balanced salt solution (BSS) is larger than the previous V4 ICL preserved in normal saline, and the V4c ICL does not expand after implantation (Table 1). Based on our previous V4 ICL sizing nomogram, an acceptable postoperative vault of about 500 μm could be achieved if the V4 ICL size is 250 μm larger than the STS. The present study was performed to identify factors related to the unexpected vault in the V4c ICL, with reference to our previous V4 ICL sizing nomogram.

**Table 1:** V4c ICL size conversion table according to the storage solution

V4c ICL size (mm) (in BSS)	V4 ICL size (mm) (in normal saline)
12.1	11.5
12.6	12.0
13.2	12.5
13.7	13.0

*Abbreviations*: *BSS* balanced salt solution

## Methods

The present study was a retrospective chart review that included consecutive patients who underwent V4c ICL implantation for the correction of myopia. Forty-three patients were enrolled, and 43 eyes were analyzed. All surgeries were conducted in the usual fashion by two surgeons (T-Y Chung and E-S Chung) at Samsung Medical Center, Seoul, Korea, between July 2013 and July 2016. The study was approved by the institutional review board of Samsung Medical Center (IRB File Number: 2016–08-092).

### Preoperative evaluation

A complete ophthalmic examination was performed before ICL implantation. The examination included uncorrected visual acuity (UCVA), best corrected visual acuity (BCVA), slit lamp examination, fundus examination, Goldmann applanation tonometry, manifest refraction and specular microscopy (SP-8000; Konan Medical, Inc., Nishinomiya, Hyogo, Japan). In addition, UBM (HiScan; Optikon, Rome, Italy) with a 35-MHz producer was performed to measure the STS [12] and the distance between the sulcus-to-sulcus plane and the anterior crystalline lens surface (STSL) [13] preoperatively. The mean keratometry (Km), pupil size, horizontal WTW and anterior chamber (AC) depth were measured by scanning-slit corneal topography (Orbscan IIz; Bausch & Lomb, Rochester, NY, USA). The AC angle and volume were measured with a Scheimpflug camera (Pentacam™; Oculus, Wetzlar, Germany).

### Determination of implantable Collamer lens

The V4c ICL is available in four overall lengths – 12.1 mm, 12.6 mm, 13.2 mm and 13.7 mm – for myopic models (VICMO) and Toric models (VTICMO). All eyes were targeted for emmetropia.

The implanted ICL sizes were individually determined with our previous V4 ICL sizing nomogram based on the STS, and the V4 ICL sizes were subsequently converted to V4c ICL sizes with a size-converting table provided by the manufacturer (Table 1). For example, if the STS was 11.75 mm, we selected 12.00 mm for the V4 ICL (pre-converted ICL size) according to our previous V4 ICL sizing nomogram. We then converted this to a V4c ICL size of 12.60 mm (converted ICL size). However, in some cases, we implanted a smaller ICL because the converted ICL size was too large. Conventionally, the implanted ICL should be larger than the STS. Thus, the “under-sizing group” was defined as having a pre-converted V4c ICL size smaller than the STS when the lens was preserved in normal saline, and the “normal-sizing group” was defined as having a pre-converted ICL size larger than the STS.

### Postoperative evaluation

After ICL implantation, patients visited our clinic 1 day, 1 week, 1 month, 6 months, and then every year after the surgery. At every visit, UCVA was assessed and Goldmann applanation tonometry and slit lamp examinations were performed to detect the development of glaucoma and cataracts. We also measured postoperative vault broadly compared with central corneal thickness via slit lamp examination. In particular, the intraocular pressure, BCVA, manifest refraction, corneal endothelial cell count (ECC) and postoperative vault were measured 6 months postoperatively and used in the analysis. The corneal endothelial cell count was measured with specular microscopy, and postoperative vault was measured precisely with anterior optical coherence tomography (Visante™ OCT; Carl Zeiss Meditec Inc., Dublin, CA, USA) [12].

### Statistical analysis

We compared postoperative vault between the normal-sizing and under-sizing groups. We also analyzed demographics (age, sex, preoperative manifest refraction spherical equivalent, BCVA, intraocular pressure and ECC), characteristics of the implanted ICL (size, dioptric power and toricity), effectiveness indexes (postoperative manifest refraction spherical equivalent and BCVA), safety indexes (postoperative intraocular pressure and ECC) and parameters related to postoperative vault (STS, STSL, WTW, preoperative Km, AC depth, AC angle, AC volume, and pupil size). Statistical analysis was performed with SPSS statistics 22.0 (SPSS Inc., Chicago, IL, USA) for Windows software. Two-sample t-tests, chi-squared tests and Fisher’s exact tests were used to determine the statistical significance of differences in parameters between the normal-sizing and under-sizing groups. A difference was considered statistically significant when the *P* value was less than 0.05.

## Results

The mean age of the patients was 28.60 ± 8.56 years (range: 18.00 to 50.00), and 6.98% of the patients (3/43) were male. The preoperative manifest refraction spherical equivalent was −9.07 ± 2.85 diopters (range: −3.00 to −15.25). Toric ICL implantation was performed in 58.14% of the patients (25/43). The mean postoperative follow-up duration was 15.43 ± 6.25 months (range: 5.17 to 26.97). Other baseline clinical characteristics are shown in Table 2.

**Table 2:** Preoperative demographics of patients undergoing V4c ICL implantation

	Normal-sizing (*N* = 19 eyes)	Under-sizing (*N* = 24 eyes)	Overall (*N* = 43 eyes)	P_Normal_Under_
Age (years)	28.79 ± 9.63	28.46 ± 7.83	28.60 ± 8.56	0.902
Sex (male, %)	1/19 (5.26%)	2/24 (8.33%)	3/43 (6.98%)	0.589 (Fisher’s exact)
ICL power (diopters)	−10.87 ± 3.16	−9.47 ± 2.98	−10.09 ± 3.10	0.144
Proportion of Toric ICL-implanted eyes (%)	10/19 (52.63%)	15/24 (62.50%)	25/43 (58.14%)	0.515 (Pearson’s chi-squared)
Preop IOP (mmHg)	17.05 ± 1.64	16.04 ± 2.90	16.49 ± 2.45	0.158
Preop MR SE (diopters)	−9.77 ± 3.11	−8.51 ± 2.56	−9.07 ± 2.85	0.154
Preop BCVA (logMAR)	0.064 ± 0.115	0.018 ± 0.052	0.038 ± 0.088	0.115
Preop ECC (/mm^2^)	2983.84 ± 769.84	2490.08 ± 1352.02	2708.26 ± 1147.42	0.140

P_Normal_Under_: *P*-values are given between the “normal-sizing” and “under-sizing” groups

*Abbreviations*: *Preop* preoperative, *IOP* intraocular pressure, *MR* manifest refraction, *S.E*. spherical equivalent, *BCVA* best-corrected visual acuity, *ECC* endothelial cell count

*Values are reported as mean ± standard deviation

The mean dioptric power of the implanted ICL was −10.09 ± 3.10 diopters (range: −3.50 to −15.75). The dioptric power of the implanted ICL did not differ significantly between the normal-sizing and under-sizing groups (−10.87 ± 3.16 diopters [range: −4.50 to −15.75] and −9.47 ± 2.98 diopters [range: −3.50 to −15.00], respectively; *p* = 0.144).

Overall, the postoperative logMAR BCVA was 0.009 ± 0.040 (range: 0.000 to 0.220) and the postoperative manifest refraction spherical equivalent was −0.177 ± 0.398 diopters (range: −1.375 to 0.500). The postoperative logMAR BCVA did not differ significantly between the normal-sizing and under-sizing groups (0.020 ± 0.600 [range: 0.000 to 0.220] and 0.000 ± 0.000 [range: 0.000 to 0.000], respectively; *p* = 0.171). The postoperative manifest refraction spherical equivalent also did not differ significantly between the normal-sizing and under-sizing groups (−0.013 ± 0.438 diopters [range: −0.875 to 0.500] and −0.214 ± 0.370 diopters [range: −1.375 to 0.250], respectively; *p* = 0.509).

The mean postoperative vault was 562.33 ± 254.29 μm (range: 60 to 1200). If acceptable postoperative vault is defined as 250 to 1000 μm, [12] 81.4% of the patients (35/43) achieved acceptable vault, 9.3% (4/43) exhibited low vault, and 9.3% (4/43) exhibited high vault. As shown in Table 3, the “Actual ICL size – STS” differed significantly between the normal-sizing and under-sizing groups (892.63 ± 262.36 μm [range: 620.00 to 1570.00] and 463.50 ± 112.75 μm [range: 274.00 to 680.00], respectively; *p* < 0.001), but postoperative vault did not differ significantly between the two groups (596.32 ± 308.64 μm [range: 60.00 to 1200.00] and 535.42 ± 204.51 μm [range: 22.00 to 1140.00], respectively; *p* = 0.442).

**Table 3:** Comparison of V4c ICL size, sulcus-to-sulcus diameter, and postoperative vault between the “normal-sizing” and “under-sizing” groups

	Normal-sizing (*N* = 19 eyes)	Under-sizing (*N* = 24 eyes)	Overall (*N* = 43 eyes)	P_Normal_Under_
Pre-converted ICL size (mm)	12.11 ± 0.36	12.13 ± 0.40	12.12 ± 0.38	0.866
Pre-converted ICL size – STS (μm)	255.79 ± 248.20	−182.33 ± 113.61	11.26 ± 286.25	0.000
Actual ICL size – STS (μm)	892.63 ± 262.36	463.50 ± 112.75	653.12 ± 288.03	0.000
Actual ICL size (mm)	12.74 ± 0.40	12.77 ± 0.44	12.76 ± 0.42	0.827
Postop vault (μm)	596.32 ± 308.64	535.42 ± 204.51	562.33 ± 254.29	0.442

Actual ICL size: V4c ICL size in balanced salt solution (BSS)

Pre-converted ICL size: ICL size in normal saline before conversion to actual V4c ICL size

P_Normal_Under_: P-values are given between the “normal-sizing” and “under-sizing” groups

*Abbreviations*: *STS* sulcus-to-sulcus diameter, *Postop* postoperative

Parameters related to postoperative vault such as the STS, STSL, WTW, preoperative Km, AC depth, AC volume, AC angle and pupil size did not differ significantly between the two groups (*p* > 0.05) (Table 4).

**Table 4:** Parameters related to postoperative vault

	Normal-sizing (N = 19 eyes)	Under-sizing (N = 24 eyes)	Overall (N = 43 eyes)	P_Normal_Under_
Postop vault (μm)	596.32 ± 308.64	535.42 ± 204.51	562.33 ± 254.29	0.442
Preop STS (mm)	11.85 ± 0.39	12.31 ± 0.41	12.11 ± 0.46	0.001
Preop STSL (mm)	0.47 ± 0.22	0.41 ± 0.17	0.44 ± 0.19	0.330
Preop WTW (mm)	11.46 ± 0.48	11.56 ± 0.39	11.51 ± 0.43	0.453
Preop Km (topo, diopters)	44.22 ± 1.35	43.10 ± 1.90	43.53 ± 1.78	0.055
Preop AC depth (topo, mm)	3.14 ± 0.29	3.24 ± 0.35	3.20 ± 0.32	0.350
Preop AC volume (Pentacam, mm^3^)	176.47 ± 39.47	193.42 ± 34.17	185.93 ± 37.14	0.139
Preop AC angle (Pentacam, degrees)	37.53 ± 6.79	38.65 ± 5.78	38.15 ± 6.20	0.564
Preop pupil size (topo, mm)	3.91 ± 0.59	4.09 ± 0.43	4.01 ± 0.51	0.240

*Abbreviations*: *Postop* postoperative, *Preop* preoperative, *STS* sulcus-to-sulcus diameter, *STSL* distance between the sulcus-to-sulcus plane and the anterior crystalline lens surface, *WTW* white-to-white corneal diameter, *Km* mean keratometry, *topo* topography, *AC* anterior chamber

Overall, postoperative ECC was 2887.81 ± 742.36/mm^2^ (range: 2358.00 to 4464.00) and postoperative intraocular pressure was 16.42 ± 2.93 mmHg (range: 12.00 to 24.00). The postoperative ECC did not differ significantly between the normal-sizing and under-sizing groups (3003.95 ± 286.28/mm^2^ [range: 2358.00 to 3472.00] and 2795.88 ± 960.34/mm^2^ [range: 2538.00 to 4464.00], respectively; *p* = 0.368). However, there was a significant difference in postoperative intraocular pressure between the normal-sizing and under-sizing groups (17.63 ± 3.45 mmHg [range: 14.00 to 24.00] and 15.46 ± 2.04 mmHg [range: 12.00 to 19.00], respectively; *p* = 0.022). This is because two eyes in the normal-sizing group exhibited an initially high vault (1200 and 1060 μm, respectively) and elevated postoperative intraocular pressure (over 21 mmHg) within the 6-month postoperative period. Therefore, we temporarily administered Alphagan-P (brimonidine 0.15%; Allergan Inc., Irvine, CA, USA), after which the intraocular pressure was maintained below 21 mmHg without medication. These patients did not progress to glaucoma. No eyes exhibited cataracts, secondary glaucoma or corneal endothelial damage during the follow-up period. There was no need for secondary ICL exchange or explantation due to complications.

## Discussion

The recently developed V4c ICL has had good outcomes and safety for the correction of myopia and astigmatism, like previous ICL models [21]. The V4c ICL also had good visual outcomes in the present study. Overall, the postoperative logMAR BCVA was 0.009 ± 0.040, and the postoperative manifest refraction spherical equivalent was −0.177 ± 0.398 diopters. We also demonstrated that the V4c ICL had good safety outcomes, without major complications such as cataracts, secondary glaucoma and corneal endothelial damage. Overall, the postoperative ECC was 2887.81 ± 742.36/mm^2^ and the postoperative intraocular pressure was 16.42 ± 2.93 mmHg.

Postoperative vault is a determinant of major complications after ICL implantation [5–10]. Acceptable levels of postoperative vault for the V4 ICL have been suggested in many previous studies. Schmidinger et al. [22] reported that, considering the decrease in vault over time after V4 ICL implantation, a minimum vault of 230 μm is necessary to prevent cataract formation. Our previous study of the V4 ICL demonstrated that if acceptable postoperative vault was defined as 250 to 1000 μm, 83.0% of patients achieved acceptable vault [12]. However, the acceptable vault for the V4c ICL is not clear. Cao et al. [21] reported that the mean vault at 6 months was 499.7 μm in the V4c group and 495 μm in the V4 group without major complications, and that the vault decreased over time for the V4c ICL. In the present study, the mean vault of the V4c ICL at 6 months was 562.33 μm, without major complications. If we assume that the acceptable vault range for V4c is the same as for V4 (250 to 1000 μm), 81.4% of patients achieved acceptable vault in the present V4c ICL study. These results are compatible with those of our previous V4 study [12].

Predicting postoperative vault in ICL implantation is challenging. Many ICL sizing nomograms for predicting the vault of V4 ICLs have been reported [11–14]. However, because of the artificial hole and BSS preservation of the V4c ICL, V4c ICL sizing may require different methods than previous ICL models.

Horizontal compression of the ICL by the ciliary sulcus is one determinant of the postoperative vault [10, 23]. Interestingly, in the present study, the vault was 596.32 ± 308.64 μm in the normal-sizing group and 535.42 ± 204.51 μm in the under-sizing group. Both groups exhibited acceptable vault, and their vault levels did not differ significantly from one another (*p* = 0.442). These results indicate that there is a buffering zone in ICL sizing. If the ICL is larger than the STS, horizontal dampening by the ciliary sulcus and vertical compression of the ICL by the iris could prevent severe overvaulting [12].

Another factor could be the insufficient inflation of the V4 ICL in the eye. In our previous study of the V4 ICL model, [12] when the average value of “ICL size – STS” was 210 μm, the mean postoperative vault was 518.6 μm. However, in the present study, the mean postoperative vault was 562.3 μm, even though the value of “pre-converted ICL size – STS” was −56 μm. According to these results, the V4c ICL can achieve acceptable vault, even though it is smaller than the previous V4 model. This can be explained by insufficient inflation of the previous V4 ICL in the eyeball, resulting from delayed enlargement of the ICL in the eyeball and morphological changes in the ICL due to its softness. In other words, V4 ICLs become thicker and larger in diameter as they inflate, and sometimes they inflate insufficiently. However, the V4c ICL is already inflated before implantation, so smaller V4c ICLs can form an acceptable vault, in contrast to V4 ICLs.

Another determinant of postoperative vault is the ICL dioptric power. Because the overall inherent vault of the ICL increases as its dioptric power increases, [10, 23] the postoperative ICL vault could increase. However, in the present study, the ICL dioptric power did not differ significantly between the normal-sizing and under-sizing groups (−10.87 ± 3.16 diopters and −9.47 ± 2.98 diopters, respectively; *p* = 0.144).

In two patients in the present study, normal sizing resulted in over-vaulting and elevated intraocular pressure. These patients tended to have smaller preoperative AC angles (36.2° and 32°, respectively) and higher implanted ICL dioptric power (−11.5 and −15.25 diopters, respectively) than the overall participants (mean AC angle, 38.15 ± 6.20°; dioptric power of implanted ICL, −10.09 ± 3.10 diopters).

Possible explanations for this abnormal over-vaulting include measurement error, individual differences in eye structure and an incorrect ICL haptic location [21]. STS-based ICL sizing is more direct and more accurate than WTW-based ICL sizing, because ICL haptic foot plates are located on the ciliary sulcus [17–20, 24]. Nonetheless, measurement error may occur in the STS method. Potential instrument error must be taken into account when the results of the present study are interpreted. Furthermore, as the ciliary sulcus is oval in shape, ICL rotation could result in vault prediction error [12]. An inappropriate ICL haptic location could also cause vault prediction error. Some of the implanted ICL haptics were not ideally placed in the ciliary sulcus, and some were even in the zonular fiber [21]. Cao et al. also reported V4c ICL over-vaulting cases, and no significant factors were found in such cases [21, 25].

In the present study, other factors such as age, sex, preoperative manifest refraction spherical equivalent, BCVA, intraocular pressure, and ECC; characteristics of the implanted ICL including size, power and toricity; effectiveness indexes including the postoperative manifest refraction spherical equivalent and BCVA; safety indexes including postoperative intraocular pressure and ECC; and parameters related to postoperative vault such as the STS, STSL, WTW, preoperative Km, AC depth, AC angle, AC volume and pupil size did not differ significantly between the normal-sizing and under-sizing groups. Therefore, other predictors of vault were not identified in the present study. Further studies are needed to reveal additional predictors of V4c ICL vault.

## Conclusions

In conclusion, we achieved acceptable vault in both the normal-sizing and under-sizing groups, and identified a buffering zone in V4c ICL sizing. It is necessary to consider the smaller size of the V4c ICL in cases susceptible to over-vaulting, such as those with shallow anterior chambers and high-dioptric-power ICLs. Further studies should include a longer follow-up period, as well as structural information on high- and low-vault cases, despite the buffering effect.
